# The Role of the Cyclin Dependent Kinase Inhibitor p21^cip1/waf1^ in Targeting Cancer: Molecular Mechanisms and Novel Therapeutics

**DOI:** 10.3390/cancers11101475

**Published:** 2019-09-30

**Authors:** Samar Al Bitar, Hala Gali-Muhtasib

**Affiliations:** Department of Biology, and Center for Drug Discovery, American University of Beirut, Beirut 1103, Lebanon; sfa28@mail.aub.edu

**Keywords:** p21, signaling pathways, cell cycle, dual role, cancer therapy, chemoresistance

## Abstract

p21**^cip1/waf1^** mediates various biological activities by sensing and responding to multiple stimuli, via p53-dependent and independent pathways. p21 is known to act as a tumor suppressor mainly by inhibiting cell cycle progression and allowing DNA repair. Significant advances have been made in elucidating the potential role of p21 in promoting tumorigenesis. Here, we discuss the involvement of p21 in multiple signaling pathways, its dual role in cancer, and the importance of understanding its paradoxical functions for effectively designing therapeutic strategies that could selectively inhibit its oncogenic activities, override resistance to therapy and yet preserve its tumor suppressive functions.

## 1. Introduction

Cell cycle checkpoint mechanisms are crucial for the protection and maintenance of genome integrity during exposure to multiple stress signals. Damaged cells can then be eliminated by apoptosis if the damage is not corrected [1]. Deregulations in these checkpoint mechanisms promote cancer development [2] and negatively influence anticancer treatment strategies [3]. One important regulator of cell cycle progression is the cyclin-dependent kinase inhibitor p21^cip1/waf1^, which is transactivated by p53 and is known to exert dual role during tumorigenesis [4].

Cell cycle progression is tightly controlled by cyclins and cyclin-dependent kinases (CDKs), the sequential activation of which allows for the initiation of and transition between different phases of the cell cycle. Initially, CDK4 and CDK6 associate with D-type cyclins, which are activated in response to mitogenic stimuli [5]. The activated cyclin/CDK complexes then phosphorylate and inactivate members of the retinoblastoma (Rb) protein family, including pRb, thus promoting progression through G1 phase and entry into the cell cycle [5]. In addition, CDK3 is thought to promote G0/G1 transition by interacting with C-type cyclins to phosphorylate pRb [6]. CDK2 activation by E-type cyclins may also be required for completion of G1 [5]. Following DNA synthesis, A- and B-type cyclins bind and activate CDK1, promoting mitosis [7].

CDKs possess several structural features that contribute to their activation. These features include cyclin-binding domain and ATP-binding domain. Most CDKs also possess activating and inhibitory phosphorylation sites [8]. The binding of inhibitory proteins, known as CDK inhibitors or CKIs, restrains the activity of CDKs [7,8]. Interestingly, CDKs and CKIs have roles beyond cell cycle regulation, as they regulate transcription, DNA damage repair, epigenetic mechanisms, and neuronal functions [8].

p21^cip1/waf1^ is a well-known potent universal CDK inhibitor that belongs to the CIP/Kip family of CKIs [9]. p21 binds and inhibits cyclin-CDK2, cyclin-CDK1, and cyclin-CDK4,6 complexes and thus, inhibits cell cycle progression during G1 and S phases [10,11]. If upregulated, p21 causes cell growth arrest at the G2 phase [12] and is required, together with p53, for sustained G2 arrest following DNA damage [13]. Notably, p21 interacts with both cyclins and CDK subunits with the kinase inhibitory domain found in the NH2 region, and thus inhibits unbound cyclins and CDKs independently. However, p21 possess higher affinity for the cyclin-associated CDK complexes. Interestingly, p21 can sometimes stabilize and activate CDK complexes depending on its abundance [14]. In addition to inhibiting CDKs, p21 interacts directly with E2F complexes and inhibits E2F transcription factor activity, leading to growth arrest [4]. Accumulating evidence also points to an important role for p21 in mediating p53-dependent regulation of cell cycle-related genes, whereby activation of p21 results in the stabilization of dimerization partner, RB-like, E2F and multi-vulval class B (DREAM) complex that is implicated in the downregulation of these genes [15,16,17]. It also binds to proliferating cell nuclear antigen (PCNA) and modulates various DNA repair processes [11]. Additionally, p21 promotes cellular senescence through various mechanisms, including gene expression regulation [18] and ROS accumulation in normal fibroblasts and in p53-negative cancer cells [11]. p21 mediates its various biological activities by sensing and responding to multiple stimuli, via p53-dependent and independent pathways. It is known to act as a tumor suppressor mainly by inhibiting cell cycle progression and allowing DNA repair. However, substantial evidence indicates that p21 has oncogenic activities that result mainly from apoptosis inhibition [9,19]. Significant advances have been made in elucidating the potential role of p21 in promoting tumorigenesis. In this review, we focus on the different mechanisms of p21 regulation and its deregulation during tumorigenesis. We further discuss the involvement of p21 in multiple signaling pathways, its dual role in cancer, and the importance of understanding its paradoxical functions for effectively designing therapeutic strategies that could selectively inhibit its oncogenic activities, override resistance to therapy and yet preserve its tumor suppressive functions.

## 2. p53-Dependent and Independent Induction of p21

### 2.1. p53-Dependent Transcriptional Regulation of p21

**Figure 1:**
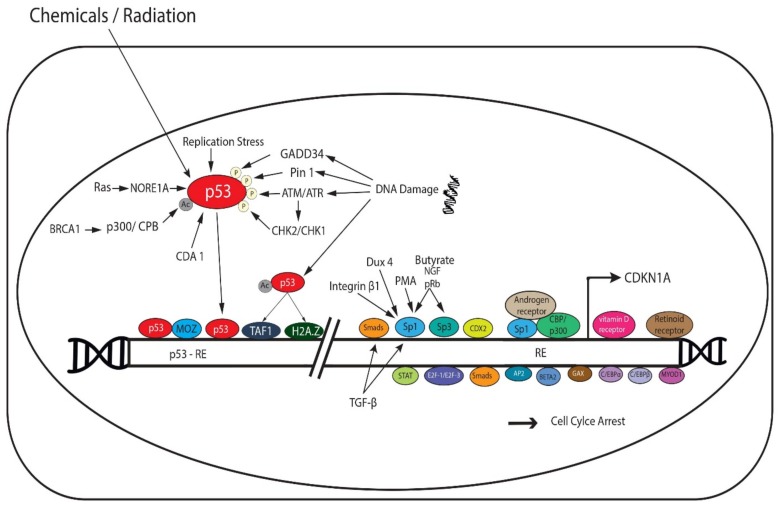
p53-dependent and p53-independent transcriptional regulation of p21. Chemicals, radiation, replication stress, and DNA damage induce the activity of p53. As a result, p53 activates the transcription of p21 by directly binding highly conserved p53-responsive elements (p53-RE) found in the p21 promotor. Acetylated p53 recruits TFIID subunit, TAF1, to the p53-RE on p21 in response to DNA damage. BRCA1-recruited p300/CREB-binding protein (p300/CBP) coactivator family mediates the acetylation of p53 and the activation of p21 gene. p53 recruits the histone variant H2A.Z to promote p21 transcription. p53 is phosphorylated and activated by ATM-CHK2, and ATR-CHK1 pathways in response to DNA damage. Pin1 and GADD34 promote the phosphorylation and stability of p53, and hence the transcriptional activation of p21 following DNA damage. Cell division autoantigen 1 (CDA1) inhibits MDM2, stabilizes p53, and thus promotes p21 transactivation. The histone acetyltransferase monocytic leukemia zinc finger (MOZ) directly interacts with p53 and induces the expression of p21. The proapoptotic Ras effector NORE1A induces p21 transcription by promoting p53 nuclear localization. pRb, Sp1, Sp3, and CDX2 transcription factors induce the expression of p21 in response to various stimuli, such as butyrate, phorbol myristate acetate (PMA) and nerve growth factor (NGF), independent of p53. Double homeobox 4 (Dux4) and Integrin β1 enhance the expression of p21 in a Sp1-dependent manner. Several nuclear receptors also activate p21 expression by binding to its RE, and include vitamin D receptors, retinoid receptors, and androgen receptors. The latter forms a complex with CBP/p300 and Sp1. Several other transcription factors also induce p21 and include signal transducers and activators of transcription (STAT), E2F-1/E2F-3, Smads, AP2, BETA2, GAX, CCAAT/enhancer binding protein-α (C/EBPα), C/EBPβ, and myoblast determination protein 1 (MYOD1). Importantly, p21 can be induced by TGF-β, in a p53-independent fashion, by activating Sp1 and Smads.

p21 is tightly regulated at the transcriptional and post transcriptional levels. p53, a tumor suppressor gene and the guardian of the genome, is a major transcriptional regulator of p21 [20]. The latter is encoded by cyclin-dependent kinase inhibitor 1A (CDKN1A) gene that is located on chromosome 6p21.2 in humans [20]. There are two highly conserved p53-responsive elements (p53-RE) in the p21 promoter to which p53 directly binds to activate p21 transcription [21]. Interestingly, a smaller protein p21B is also produced by the p21 gene [22] from an alternative promotor (P2 promotor) that also contains a p53-RE [21]. p53 activity is induced by multiple extrinsic signals, such as chemicals and radiation, and by several intrinsic stress signals, such as replication stress and DNA damage. Thereby, p53 responds by transcriptionally upregulating the appropriate gene targets, including p21 (Figure 1) [23]. p53 post-translational modifications, including acetylation and phosphorylation, have been shown to enhance its stabilization and activation in response to stress stimuli and activation of DNA damage response and repair pathways [4,24,25]. In response to DNA damage, acetylated p53 recruits TFIID subunit, TAF1, to the p53-RE on p21 [26]. In addition, p53 acetylation, at the K164 residue in the DNA-binding domain by p300/CREB-binding protein (p300/CBP) coactivator family, is important for the activation of p21 gene [27]. DNA damage-induced p53 mediates transcription initiation of p21 gene by recruiting the histone variant H2A.Z that renders the promotor region more permissive to transcription [28]. Following DNA damage, p53 is phosphorylated by Ataxia telangiectasia mutated (ATM) and RAD3-related (ATR). ATR and ATM phosphorylate and activate two members of the serine/threonine kinase family, CHK1 and CHK2, respectively. In turn, CHK1 and CHK2 further phosphorylate and activate p53 [29].

Numerous cellular factors interact with p53 and activate it to regulate p21 transcription. A good example is BRCA1, which recruits p300/CPB that acetylates and stabilizes p53 [30]. Pin1 has been also shown to regulate p53 stability and therefore the transcriptional activation of p21 in response to DNA damage [31]. This occurs by inducing conformational changes in p53 and thereby facilitating its phosphorylation at Ser-33 and -46 [32]. GADD34, a DNA damage-inducible protein, promotes the phosphorylation of p53 and subsequent p21 transcription [4]. Cell division autoantigen 1 (CDA1) enhances stabilization of p53 by inhibiting MDM2, a ligase that mediates p53 degradation, and thus promotes p21 transactivation [33]. Moreover, the histone acetyltransferase monocytic leukemia zinc finger (MOZ) directly interacts with p53 and induces expression of p21 after recruitment of the MOZ-p53 complex to the p21 promotor [34]. The proapoptotic Ras effector NORE1A induces p21 transcription by promoting p53 nuclear localization [35]. A member of the Krüppel-like transcription factor (Klf) family that regulate cellular proliferation, KLF4, mediates damage-induced p53 response by upregulating p21 expression and inducing cell cycle arrest at G1 phase [36].

### 2.2. p53-Independent Transcriptional Regulation of p21

Several other molecules induce the expression of p21 independent of p53 (Figure 1). These include transcription factors that use responsive elements in the proximal p21 promotor to induce the expression of p21 in response to various stimuli, such as butyrate, phorbol myristate acetate (PMA) and nerve growth factor (NGF) [23]. Examples include pRb [37], Sp1, and Sp3 [38]. Double homeobox 4 (Dux4), a transcription factor that induces G1 phase arrest, enhances the expression of p21 in a Sp1-dependent manner [39]. CDX2, a member of the caudal-related homeobox gene family, transactivates p21 promotor [40]. Moreover, integrin β1 was shown to enhance transcription of p21 by recruiting Sp1 to p21 promotor region [41]. Several nuclear receptors also activate p21 expression by binding to its RE, independent of p53. These include vitamin D receptors, retinoid receptors, and androgen receptors [38]. The latter is thought to form a complex with CBP/p300 and Sp1 [38]. A member of the Klf family, Klf6, is recruited to p21 promotor and is acetylated by p300-CREBBP to induce p21 transcription [42]. Several other transcription factors also induce p21, these factors were reviewed by Gartel and Tyner [23] and include signal transducers and activators of transcription (STAT), E2F-1/E2F-3, Smads, AP2, BETA2, GAX, CCAAT/enhancer binding protein-α (C/EBPα), C/EBPβ, and myoblast determination protein 1 (MYOD1) [23]. Importantly, p21 can be induced by extracellular antiproliferative signals including TGF-β, in a p53-independent fashion, by activating several transcription factors, such as Sp1 and Smads [43].

## 3. Post Transcriptional Regulation of p21

### 3.1. Phosphorylation, Stability, and Subcellular Localization of p21

**Figure 2:**
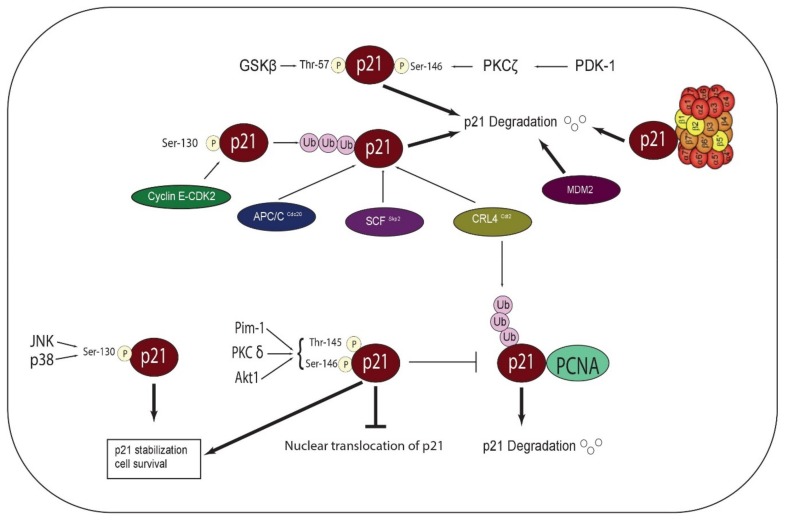
Post-transcriptional regulation of p21. Pim-1, PKC, and Akt1 kinases phosphorylate Thr-145 and Ser-146 sites on p21 protein. Phosphorylation at Thr-145 promotes cytoplasmic translocation and stability of p21. Ser-146 phosphorylation mediated by Akt1 enhances p21 stabilization and promotes cell survival. Phosphorylation of p21 at either site disrupts the interaction between p21 and PCNA, thus inhibiting CRL4-mediated ubiquitination and degradation of p21. Phosphorylation at Ser-146 by PKCδ increases p21 stability, whereas phosphorylation of the same serine by PDK1-activated PKCζ results in p21 degradation. Similarly, glycogen synthase kinase-3 (GSKβ) phosphorylates Thr-57 and induces p21 degradation. JNK and p38 phosphorylate p21 at Ser-130 and increase its stability. Cyclin E- CDK2 complex phosphorylates p21 at Ser-130, resulting in the ubiquitin-dependent degradation of p21. Cell cycle-related ligases mediate ubiquitination and degradation of p21, and include Anaphase Promoting Complex/Cyclosome and its activator Cdc20 (APC/CCdc20), SCFSkp2, and CRL4Cdt2. p21 can directly bind the C8 alpha-subunit of the 20S proteasome, mediating proteasome assembly and ubiquitylation-independent degradation of p21. Strikingly, MDM2 mediates the degradation of p21 independent of its ubiquitin E3 ligase activity.

Phosphorylation of p21 protein confers an additional level of regulation, and subsequently affects the stability and subcellular localization of p21. The ability of p21 to regulate cell cycle and other processes relies largely on its subcellular localization. Thr-145 and Ser-146 sites are phosphorylated by Pim-1, PKC, and Akt1 kinases. Phosphorylation at Thr-145 inhibits the nuclear translocation of p21 in breast cancer cells and promotes p21 stability (Figure 2) [20]. Ser-146 phosphorylation mediated by Akt1 enhances p21 stabilization and promotes cell survival [31]. It has been also documented that phosphorylation of p21 at Thr-145 or Ser-146 disrupts the binding of p21 with PCNA [4]. Phosphorylation at Ser-146 by different PKCs has shown conflicting results and has been a matter of debate. For example, phosphorylation at Ser-146 by PKCδ increases p21 stability [44]. Conversely, phosphorylation at Ser-146 by PDK1-activated PKCζ results in p21 degradation [45]. Similarly, glycogen synthase kinase-3 (GSKβ) phosphorylates Thr-57 and decreases the stability of p21 by inducing its degradation [38]. Interestingly, cyclin E- CDK2 complex phosphorylates p21 at Ser-130, which results in the ubiquitin-dependent degradation of p21 [38,46]. On the other hand, JNK and p38 phosphorylate p21 at Ser-130 and increase its stability [20].

### 3.2. Ubiquitin-Dependent Degradation of p21

Ubiquitin-dependent proteasomal degradation confers an important regulatory mechanism for p21 stability, and thereby for cell cycle progression control. p21 is targeted for ubiquitylation and degradation by multiple cell cycle-related ligases at different stages of the cell cycle [20]. For example, a study has shown that Anaphase Promoting Complex/Cyclosome and its activator Cdc20 (APC/C^Cdc20^) control the degradation of p21 in prometaphase, and thus promote the full activation of CDK1, which is needed for the progression of mitotic events [47]. Other studies have shown that SCF^Skp2^ and CRL4^Cdt2^ target p21 for degradation during S phase [46,48]

### 3.3. Ubiquitin-Independent Degradation of p21

Ubiquitin-independent degradation of p21 has been also observed in a number of studies [49]. It has been established that p21 can directly bind the C8 alpha-subunit of the 20S proteasome, resulting in the assembly of the proteasome, independent of ubiquitylation [50]. Strikingly, MDM2 mediates the degradation of p21, independent of its ubiquitin E3 ligase activity [51].

## 4. Deregulation of p21 in Cancer

Being a tumor suppressor, p21 is usually downregulated in many types of cancer and contributes to poor prognosis of patients and decreased overall survival [19,38]. p21-deficient mice show a defective G1 checkpoint control, but do not develop spontaneous tumors [52]. Although p21 mutations are rare, they have been reported in a number of human cancers, including Burkitt’s Lymphoma [53], melanomas [54], thyroid carcinomas [55], and breast cancer [56].

The decreased expression of p21 correlates with mutations in the genes that activate and repress CDKN1 transcription. These genes undergo gain of function or upregulation and loss of function or downregulation, respectively [38]. Given that p21 is a direct target of p53, loss of p53 activity leads to downregulation of p21 in many types of cancer [20,38]. The most known suppressor of p21 transcription is c-Myc [57]. The latter is a proto-oncogene whose overexpression during cancer results in the activation of other oncogenes and suppression of many growth arrest genes including p21 [57]. It has been documented that c-Myc mediates estrogen-dependent breast cancer cell proliferation by targeting p21 and thus contributing to estrogen resistance to therapies in ERα-positive breast tumors [58]. The transcription factor AP4 has been also shown to repress p21 expression by binding to recognition motifs in the promotor region of p21 [59]. Interestingly, AP4 is induced by c-Myc and is required for c-Myc-mediated cell cycle reentry of breast cancer cells. Moreover, the induction of c-Myc and repression of p21, possibly through the activation of the β-catenin/TCF-4 complex that controls c-Myc activity [60], has been postulated to play a critical role in colorectal cancer [61]. The mechanistic basis of p21 repression by c-Myc has been addressed by many studies and reviewed by Jung et al. [20]. It has been suggested that the binding of c-Myc to Sp1/Sp3 transcription factors on the proximal promotor region of p21 mediates this repression. Association of c-Myc with and inhibition of Miz1, an initiator sequence binding factor, is known to mediate p21 repression by c-Myc [20].

Tbx2, which is amplified in some breast cancers, regulates senescence and oncogenesis, at least in part, through the regulation of p21 expression [62]. Histone deacetylase 4 (HDAC4) promotes gastric cancer progression by downregulating p21 [63]. Human MORC2 (microrchidia family CW-type zinc-finger 2), a chromatin regulator during DNA-damage response and a transcriptional repressor, was found to be upregulated in gastric cancer, whereby it represses p21 expression by recruiting HDAC1 to p21 promotor, in a p53-independent manner [64]. A study has reported that cell division cycle 27 (CDC27), a core subunit of the anaphase-promoting complex/cyclosome, downregulates p21 mRNA levels to promote proliferation of colorectal cancer cells [65].

The interaction of p21 with PCNA mediates the ubiquitination and degradation of p21 by a component of the CRL4 E3 ubiquitin ligase family, known as CRLCDT2 or DTL in UV-irradiated cells and neoplastic cell lines [66,67].

Loss of cell cycle control promotes tumorigenesis and reflects a crucial hallmark of cancer [2]. In particular, most human cancers manifest an impaired G1/S checkpoint control, most commonly caused by mutations in p53, a major regulator of G1/S checkpoint [68]. Interestingly, it has been documented that p21 is important for p53-dependent G1 arrest in human cancer cells [69]. Due to its significant implication in cell cycle regulation, p21 is often targeted in many types of cancers to counteract or enhance the tumor suppressor activities it exerts. For example, Inhibitor of Growth 2 (ING2) is a tumor suppressor that is downregulated in human cancers. ING2 is known to activate p21 expression by modifying the methylation patterns at the p21 promotor; however, the loss of ING2 in cancer results in decreased expression of p21 and increased progression from G1 to S phase [70].

In contrast, the overexpression of p21, together with an observed oncogenic activity, has been detected in many human cancers associated with poor prognosis, including breast, cervical, prostate cancer, gliomas, and acute myeloid leukemia (AML). It has been shown that Ras oncogene, which is frequently mutated and activated in human cancers, induces p21 promotor partly through the transcription factor E2F1 in a p53-independent fashion [71]. Overexpression of cytoplasmic p21 has been found to exert antiapoptotic effects and confer chemotherapeutic resistance in tumor cells [72]. In fact, p21 can act as either a tumor suppressor or an oncogene depending on cell type, stress factors, and p53 state. The latter is the major determinant of p21 properties and activities, as p21 functions to inhibit carcinogenesis in a p53-proficient environment while it promotes tumorigenesis in a p53-deficient environment [11]. It is now well established that p21 exerts different effects on tumorigenesis when localized within different subcellular compartments. When in the nucleus, p21 functions as a tumor suppressor. In contrast, when localized and overexpressed in the cytoplasm, p21 functions as an oncogene, as evidenced from immunohistochemistry studies using human tumor cells and tissues [38,73]. Cytoplasmic localization of p21 is associated with cell growth and survival, by promoting the assembly of the D-type cyclins with CDK4 and CDK6 and inhibiting apoptosis, respectively [38]. The assembly and activation of this complex by p21 is dependent on the abundance of p21 in vitro and in vivo [14]. p21 in the cytoplasm may also act as a chaperone for cyclin E to initiate G1/S progression. It is suggested that when p53 is damaged or lost, cytoplasmic p21 may exert the above-mentioned oncogenic effects, in addition to facilitating cell migration [11]. On the other hand, nuclear localization of p21 favors its tumor suppressive activities by inhibiting cell division and growth. p21 can also downregulate some DNA repair pathways in the nucleus and induce senescence [11,38,74]. Importantly, the relation between p53 status and p21 subcellular localization is not fully understood. So far, two studies have shown conflicting results in this regard. In one study, the high cytoplasmic levels of p21 were found to be associated with high p53 and cyclin B levels in breast cancer [75]. In another study, the nuclear localization of p21 was associated with p53 detection and PCNA expression in multiple myeloma [76]. Others have reported a correlation between p21 downregulation and increased p53 detection, but the subcellular localization of p21 was not mentioned [38]. Taken together, p21 can exert opposing roles in cancer by directly or indirectly regulating the activity of oncogenes and tumor suppressors. In the nucleus, p21 generally inhibits cyclin-CDK complexes, thus leading to the direct inhibition of cell proliferation. Nuclear p21 has indirect negative effects on cell cycle-related genes and oncogenes, such as c-Myc. It also interferes with the activities of several transcription factors. p21 also represses the transcription of anti-apoptotic genes. In contrast, cytoplasmic accumulation of p21 results in cell cycle progression and direct apoptosis inhibition through interfering with the activity of several caspases. Overall, p21 confers a broad range of interaction with many proteins and critical players in tumorigenesis, which partly explains its dual function in the cytoplasm versus the nucleus, depending on the proteins available in each compartment and the type of interactions that occur. The identification of novel targets would reveal new functions for p21 in each compartment and would provide a better understanding of the role of its cytoplasmic re-localization in promoting oncogenesis.

## 5. Functions of p21

### 5.1. Role of p21 in Cell Cycle

Growth stimuli sequentially activate CDKs to promote cell cycle. One of the major events that occur early during the cell cycle is pRb phosphorylation by cyclin D-CDK4/6. Phosphorylation of pRb is indispensable for the G1/S transition as it releases the sequestered E2F transcription factors and allows for the transactivation of genes important for the exit from G1 and the entry into S phase. CyclinE/CDK2 complex is activated to further phosphorylate pRb and ensure complete separation of E2F from the hyperphosphorylated pRb [77]. CDK3 associates with cyclin C to phosphorylate pRb, preceding pRb phosphorylation by CDK4, CDK6, and CDK2. This facilitates the exit of cells from the G0 phase and entry into G1 phase [6].

The role of p21 in cell cycle control is well established. p21 can directly inhibit cyclin-dependent kinase activities by interacting with their N-terminal domains or indirectly by interfering with the phosphorylation of CDK1 and CDK2 [38]. p21 binds the cyclin subunit through a conserved cyclin-binding motif 1 (Cy1), which is present in the N-terminal region. Additionally, p21 binds through redundant weak motif, Cy2, in its C-terminal region. These binding motifs are indispensable for p21-mediated inhibition of cyclin-CDK complexes. p21 interacts with CDK through a CDK-binding motif or kinase site in the N-terminal region for optimal inhibition [19]. Interestingly, p21 shares similar structural Cy motifs with other regulators of cell cycle, which enables it to compete with and disrupt the interactions between these regulators and the CDK-cyclin complexes. For example, p107 and p130, two structurally similar members of the pRb family, have a cyclin-binding domain Cy1 that is distinct from the E2F-binding domain, which allows them to interact with and inhibit cyclinE/CDK2 or cyclinA/CDK2 complexes [38]. Cdc25A is a phosphatase that activates cyclinE/CDK2 complex through association with the complex and dephosphorylation of CDK2, thereby mediating G1-S transition. It has been shown that cdc25A has a cyclin binding motif that is similar to Cy1. The presence of a similar motif in the N terminal regions of p21 and cdc25A allows for a competitive antagonism in the regulation of cell cycle progression [38]. p21 mediates cell cycle arrest in G1 phase primarily by inhibiting the activity of cyclinA/CDK2 and cyclinE/CDK2 [10]. As a result, cyclin/CDK2 complexes cannot phosphorylate pRb, and thus E2F transcription factors remain sequestered and unable to promote entry into S phase [38]. In line with this finding, mouse embryonic fibroblasts (MEFs) from mice lacking p21 have shown an impaired G1 checkpoint control [52]. In response to γ-irradiation, p21 can also inhibit CDK1 and prevent G1/S transition in CDK2−/− cells which is due to the ability of CDK1 to substitute for CDK2 [78]. Several reports indicate that p21 mediates G1/S checkpoint control by interacting with the central region of PCNA [38,79]. This interaction, which is mediated by the C-terminal domain of p21, displaces DNA replication enzymes from PCNA and directly blocks DNA synthesis [38] However, these findings were limited to in vitro models, as p21 is usually downregulated in vivo during the S phase [80].

Previous work has identified the involvement of p21 in G2 checkpoint control. It is important to note that p21 has low affinity for cyclin B/CDK1 in comparison to other cyclin/CDK complexes [81]. However, mechanisms of p21-mediated G2 phase arrest include inactivating cyclinB/CDK1 complexes [13] by inhibiting CDK-activating kinase (CAK) and thereby inhibiting CDK-1 activating Thr^161^ phosphorylation [82]. p21 has been also demonstrated to associate with and inactivate cyclin A-CDK1/2 complexes. p21 promotes the long-term inactivation of cyclinB1/CDK1 complexes by nuclear sequestration and inhibition of CAK and cdc25, in response to genotoxic stress [81].

Studies on p21 deficiency highlight the role of p21 in normal mitotic processes. Loss of p21 results in centriole overduplication and consequently an abnormal increase in centrosome number in tumor cells [13]. p21 is important for controlling CDK1 activity during mitosis since the deficiency of p21 in tumor cells has been shown to cause multiple mitotic defects including prolonged mitosis, abnormal chromosomal segregation and cytokinesis [83]. On the other hand, induced overexpression of p21 inhibits mitosis and inhibits CDK1/cyclin B1 kinase activity [82]. Similar to p21 loss, p21 overexpression also results in polyploidy; however, it also leads to partial inhibition of CDK1 activity [12].

Nonetheless, it has been suggested that p21 can promote cell growth possibly by functioning as an adaptor protein that induces the assembly, nuclear translocation, and activation of cyclin D/CDK4 in human tumor cells, resulting in cell proliferation [14,84]. These studies highlight a potential conflicting role of p21 in promoting oncogenesis through activation of cell cycle regulators [84]. The opposite cell cycle regulatory roles of p21 seem to depend on p21 expression level, whereby at low levels p21 promotes cyclin D1/CDK4 complex assembly and activation. The positive interaction of p21 with these complexes may serve to titrate p21 and free cyclin/CDK2 complexes from the inhibitory effect of p21 during cell cycle progression. In contrast, at high levels, p21 exerts inhibitory effects on the kinase activity of the CDK4 complex. These findings were further confirmed in vivo as the absence of p21 was found to contribute to reduced cyclin D-dependent kinase activity. However, the requirement of p21 for cell cycle progression remains unclear and requires further investigation in other cell systems and animal models [52,85], especially that different cell types show different levels of p21 protein in vivo [86], and during different phases of the cell cycle [87]. Future in vivo investigations of the dual role of p21 in cell cycle progression will help determine its role in the different hallmarks of cancer.

### 5.2. Role of p21 in Apoptosis

Multiple studies shed light on the potential role of p21 in modulating apoptosis and tumorigenesis. p21 is a negative regulator of p53-dependent and p53-independnet apoptosis in cancer cells [88]. Several mechanisms have been described for apoptosis inhibition by p21. These include direct interaction with and inhibition of several apoptosis regulatory proteins, such as protease precursors and kinases [89,90,91]. p21 interacts with procaspase 3, through its first N-terminal 33 amino acids, preventing the conversion of the inactivated procaspase 3 into its active form, thus inhibiting Fas-mediated apoptosis [89]. p21 also mediates the repression of caspase 2 in a p53-dependent manner [92]. On the other hand, p21 is cleaved by caspases during DNA damage-induced apoptosis [90]. Reports show that cytoplasmic p21 inhibits apoptosis signal-regulating kinase 1 (ASK1) and stress-activated MAP kinase cascade and acts as an inhibitor of apoptosis [91]. p21 also induces genes with known mitogenic or anti-apoptotic effects [18].

DNA damage results in two scenarios: Apoptosis or p21-dependent cell cycle arrest. In cases where p53-dependent p21 induction is repressed or p21 protein is inactivated by caspase-mediated cleavage, p53-dependent apoptosis is the end result [11]. Following exposure to DNA-damaging agents, human cancer cell lines undergo p21-mediated cell cycle arrest. However, the subsequent cleavage of p21 by caspase 3 results in apoptosis induction [93]. p53 point mutations that affect p53’s ability to transactivate p21 resulted in a more potent induction of apoptosis as compared to wild-type p53 [94].

c-Jun induces p53-dependent apoptosis partly by negatively regulating the association of p53 with p21 promotor. In contrast, c-Jun-deficient cells undergo p21-mediated cell cycle arrest in response to UV [95]. Interleukin 3 (IL-3) was shown to play a role in the induction of p21 and cell cycle arrest in irradiated Baf-3 murine hematopoietic cells with wild-type p53. This was further supported by the observation that the radiation of these cells in the absence of IL-3 resulted in weaker induction of p21 and thus, cell death [88,96]. p21 antisense therapy radiosensitizes human colon cancer to apoptosis [81].

Gartel and Tyner [88] reviewed the effect of several drugs on p21 and consequently, on cellular response and apoptosis. For example, when administered with the DNA-damaging drug Adriamycin, the immunosuppressive agent triptolide enhanced apoptosis by upregulating p53 expression and repressing p21 transcription. Adriamycin induces p53-dependent and -independent apoptosis; however, the expression of p21 counteracts the effect of this drug. This is supported by the fact that Adriamycin-treated HCT116 human colon carcinoma cell line undergoes cell cycle arrest whereas Adriamycin-treated p21-null HCT116 cells undergo apoptosis. Moreover, inhibition of CDK2 activity seems to be a mechanism by which p21 negatively regulates apoptosis. Overexpression of p21 in human osteosarcoma cells with wild-type p53 protects cells from apoptosis induced by the cancer chemotherapeutic drug etoposide [88].

The effects of oxidative stress on p21 was also reviewed [88]. Hydrogen peroxide (H_2_O_2_) can activate two different pathways: p53-dependent apoptosis and p53-dependent and -independent induction of p21 that protects cells from apoptosis. Nitric oxide and hyperoxia are known to induce p53-dependent and -independent activation of p21 and cell cycle arrest in macrophages, vascular smooth muscle cells, and HCT116 cancer cells. Interestingly, antisense p21 impairs nitric oxide-induced G1 arrest and sensitizes cells to apoptosis. Similarly, hyperoxia induces apoptosis in HCT116 cells, in the absence of p53 or p21. p21 seems to protect cells from oxidative stress, whereby it increases survival of mice, following hyperoxic lung injury [97].

p21 has been also shown to negatively regulate p53-independent apoptosis. Signals including TGF-β, TNF-α, IFN-γ, and histone deacetylase inhibitors may simultaneously induce both p53-independent apoptosis and p53-independent upregulation of p21 [23]. TGF-β induces cell cycle arrest or apoptosis to inhibit cellular proliferation. The induction of p21 in TGF-β-treated colon cancer cells correlated with reduced cyclin E-associated kinase activity in vitro and reduced pRb phosphorylation in vivo, suggesting that p21 sensitizes cancer cells to TGF-β-induced cell cycle arrest [98]. However, p21 mediates resistance to TGF-β-induced apoptosis [88]. Like TGF-β, TNF-α induces p21 expression and apoptosis in malignant Ewing tumor and breast cancer cells in a NF-κB-dependent manner. p21 antisense oligonucleotides sensitizes cells to TNF-α-mediated apoptosis [88]. IFN-γ induces p21 in a STAT1-dependent fashion, which in turn induces G1 arrest and inhibits apoptosis. Histone deacetylase induce p21 through Sp1 binding sites in the p21 promotor, which causes cell cycle arrest and/or apoptosis in human colon cancer and leukemia cells [88].

Interestingly, p21 has a conflicting role in promoting apoptosis. This finding is based on several reports showing that p21 overexpression enhances apoptosis response to cisplatin in glioma cells and ovarian carcinoma cells in vitro [99]. Moreover, p21 enhances MAPK-dependent apoptosis that is stimulated by bile acid in hepatocytes [100]. p21 also promotes ceramide-induced apoptosis by increasing the expression of the proapoptotic protein Bax [101]. CD95/Fas signaling induces p21 to promote apoptosis in T lymphocytes [102]. Additionally, a proapoptotic function of p21 has been found in differentiating granulocytes, whereby IL-3 was reported to inhibit p21-dependent apoptosis [103]. Thus, p21 may have pro-or anti-apoptotic effects depending on cell type, stress factors, and nature of apoptotic stimulus. Moreover, p21’s response to different drugs depends on the mode of action of the drug and on cellular context. As discussed above, the subcellular localization of p21 is a critical determinant of its role in apoptosis, whereby cytoplasmic p21 is free to interact with and inhibit positive regulators of apoptosis [91]. In addition, p21-induced apoptosis appears to be an alternative or a complementary process to cell cycle arrest following DNA damage and may also depend on p53 status. Thus, it would be challenging to infer whether p21 has a direct role in apoptosis, apart from its direct interaction with apoptotic proteins in the cytoplasm. On the other hand, p21 accumulation in the nucleus correlates with enhanced apoptosis in response to specific drugs [99]. However, it is not clear whether p21 is essential for apoptosis. Many studies have shown induction/repression of p21 in response to various apoptotic and anti-apoptotic stimuli, however, few studies have reported or addressed the molecular pathway(s) affected downstream of p21. Gene expression profiling and protein-protein interaction studies, that account for p53 status in the studied model systems, are recommended to gain a global picture of the multiple functions mediated by p21 and to explore the possible molecular mechanism by which p21 affects apoptosis.

### 5.3. Role of p21 in DNA Repair

Evidence from multiple studies indicates that p21 has a dual role in DNA repair processes [104,105,106]. p21 allows DNA repair by inhibiting cell cycle progression and apoptosis; however, it is well established that p21 interacts with PCNA and competes with several DNA repair components associated with PCNA [107]. p21 interferes with PCNA-DNMT1 binding, which is required for DNA repair and synthesis [19]. Moreover, p21 or p21-derived PCNA interacting peptide inhibit mismatch repair activity and PCNA-dependent base excision repair [38]. p21 also inhibits the interaction between PCNA and translesion DNA synthesis (TLS) enzyme, thereby modulating DNA replication and repair following UV irradiation [104]. p21 was also shown to inhibit PCNA ubiquitination that is required for the PCNA-dependent repair after UV irradiation [105]. On the other hand, the UV-triggered p21 degradation and removal from replication forks is crucial for facilitating damaged-DNA replication and preserving genomic stability [11].

The role of p21 in nucleotide excision repair (NER) regulation is controversial [108]. Defects in NER genes lead to a rare genetic disorder, known as xeroderma pigmentosum, and consequently skin cancer [109]. The xeroderma pigmentosum group E gene product DDB2, which is also a component of the CRL4 E3 ubiquitin ligase complex, recognizes DNA damage in NER and promotes p53 degradation and the consequent downregulation of p21 in UV-irradiated cells [110]. Interestingly, p21 upregulation has been shown to inhibit NER activity in DDB2−/− mice, whereas its deletion restores NER activity, further confirming the role p21 in repressing NER activity [110]. In contrast, it was reported that p21 did not inhibit NER in vivo [104] and that p21−/− cells showed NER deficiency [111]. Another mechanism by which p21 regulates NER is through binding to p300 acetyl transferase, disrupting the association between p300 and PCNA, and promoting DNA repair [106]. p21 modulates the interaction between p300 and XPG, the 3′-endonuclease that mediates the incision/excision step, and thus, influences the acetylation of XPG [112].

p21 is also involved in double-strand break (DSB) repair pathway, whereby it colocalizes with proteins involved in DNA repair, such as Rad50, MRE11, and PCNA at DNA damage sites induced by heavy ions [113]. p21 participates in the two major pathways that repair DSB: The error-free mechanism based on Homologous recombination (HR) and the error-prone process, nonhomologous DNA-end-joining (NHEJ). p21−/− cells exhibit reduced replication-coupled HR and increased MRE11 nuclear foci and CDK-mediated BRCA2 phosphorylation [114]. p21 foci accumulate at irradiated DSB sites, which are mainly repaired by NHEJ. The accumulation of EGFP-p21 was detected in both normal and transformed cell lines and was independent of p53 and NHEJ factors Ku70, Ku80, and DNA-PK. Instead it occurred in a PCNA-dependent fashion [115].

It has been reported that the downregulation of the major DNA repair factor histone H2AX is mediated by Her2/NeuT oncogene and involves the p21-CDK-Rb pathway, resulting in suppression of DNA repair while promoting DNA instability in Her2-positive cancer [116].

Taken together, the abovementioned findings show a direct role of p21 in DNA repair processes via regulating PCNA interactions with multiple DNA repair factors. Others have suggested that p21 promotes DNA repair through inducing cell cycle arrest during low levels of DNA damage, whereas high levels of DNA damage result in p21 degradation and apoptosis induction [81]. Thus, the extent of genotoxic stress and the pathways affected downstream may determine the role of p21 in genome stability. Further exploration of the different DNA repair proteins that interact with p21 may help elucidate the detailed mechanisms involving p21 during DNA damage in physiological systems. Importantly, a computational analysis may be essential for distinguishing direct and indirect p21-protein interaction networks and reconstructing signaling pathways involving p21 [117]. Such analyses may provide a new perspective pertaining to the role of p21 in DNA repair processes.

### 5.4. Role of p21 in Transcriptional Regulation

p21 can have positive or negative effects on transcriptional regulation in response to DNA damage [118,119,120]. p21 can repress transcription by inhibiting cyclin-CDK complexes, and thus inhibit phosphorylation of pRb and repress E2F-dependent transcription of many genes [118]. It can also function as a transcriptional cofactor by directly binding and regulating the activity of several transcription factors, including NF-κB, E2F, and estrogen receptors [118,121,122]. p21 also negatively regulates expression levels and stability of p53 [123]. It has been shown that p21 associates with STAT3; the overexpression of p21 in vivo reduced the transcriptional activity of STAT3 without affecting its DNA binding activity [124]. Moreover, p21 can repress E2F-dependent transcription, not only through CDK inhibition but also through direct interaction and inactivation of E2F transcription factors [125]. p21 suppresses c-Myc-dependent transcription by binding to its N-terminal region and interfering with c-Myc-Max complex formation. The binding of c-Myc with p21 competes with PCNA and modulates DNA synthesis and replication [126]. It was also reported that p21 down regulates *myc* and *cdc25A* genes by binding to the promotors of these genes, after being recruited together with STAT3 and E2F in response to DNA damage. The recruitment of p21 is associated with downregulation of histone H4 acetylation and inhibition of p300 histone acetylase recruitment [127]. p21 and acetyltransferase p/CAF regulate TGF-β transcriptional activity on several tumor-promoting genes by regulating Smad3 acetylation and DNA binding. The regulatory mechanism mediated by p21 was associated with tumor cell invasion and lymph node involvement in tissues obtained from breast cancer patients [128].

It has been reported that p21 inhibition of the polo-like kinase 1 (PLK1) is mediated in part by two promotor elements, cell cycle-dependent element (CDE) and cell cycle genes homology region (CHR), whereas the inhibition of topoisomerase IIα (TOPO IIα) promotor by p21 is mediated through CDE and not CHR. These DNA sequences are implicated in the regulation of cell cycle-dependent transcription and have been identified in the proximity of the initiation start site in the promotors of genes inhibited by p21, such as *cyclin B1* and *PLK1* [129]. CDE was also found in most of the promotors of the S-phase and mitotic control genes regulated by p21 in human leukemia cells and primary keratinocytes. The identified genes included cyclin E2 gene (CCNE2), CDK2, KIF4A, and WEE [130].

p21 is a negative transcriptional regulator of Glucocorticoid-induced tumor necrosis factor receptor (GITR), which exerts anti-apoptotic functions in T lymphocytes and keratinocytes. By downregulating GITR, p21 enhances the UVB-induced apoptosis and response to DNA damage in skin keratinocytes [131].

p21 can also positively regulate gene transcription mediated by several transcription factors. It enhances transcriptional activation by Estrogen receptor (ERα) through two mechanisms [119,120]. The first is through inhibition of CDK2 activity, which subsequently alleviates the CDK2-mediated block on CREB-binding protein (CBP), thus enhancing CBP histone acetyltransferase (HAT) activity and amplifying ERα activity [120]. The second is through associating with CBP and ERα, which facilitates the recruitment of CBP to the receptor and regulates interactions responsible for transcriptional activation of ERα [122].

p21 regulates transcription through a third mechanism that involves interaction with co-activators, such as p300 or CBP [119]. It negatively regulates *Wnt* gene transcription by binding to *Wnt4* gene promotor in association with E2F-1 and inhibiting the recruitment of p300 to the promotor, resulting in histone hypoacetylation and transcription repression [132]. The binding of cyclin/CDK complexes with p300 inhibits the interaction between p300 and NF-κB, thereby suppressing NF-κB-dependent transcription. On the other hand, p21 alleviates this inhibition by restricting the activity of cyclin/CDK complexes and promoting p300-mediated transcription [119]. p21 induces the transcriptional activity of p300 through indirect interaction with the CRD1 motif, a discrete inhibitory domain within p300 and CBP [133]. p21 upregulation has been reported to reduce p300 and contribute to the down regulation of the DNA methyltransferases DNMT1, hence regulating DNA methylation in mammalian cell division [134].

p21 regulates many other genes involved in cell division, senescence, and aging [18]. Examples include t-TGase, which has been implicated in cell differentiation, carcinogenesis, apoptosis, and aging. p21 induction of cathepsin B, PAI-1, fibronectin, and N-acetylgalactosamine-6-sulfate sulfatase has been associated with arthritis [18]. p21 also represses the transcription of high mobility group box 2 (HMGB2) during radiation-induced senescence through the ATM-p53-p21 DNA damage pathway. Notably, HMGB2 is a protein involved in transcription, chromatin remodeling, recombination, and senescence [135].

It has been also shown that p21 mediates inflammation suppression by reducing the expression of TNF-α and IL-1β in macrophages [136]. In contrast, no inflammatory response was observed in p21-knock out mice in lung inflammation induced by cigarette smoke [137].

As part of the p53-DREAM pathway, p21 has been shown to indirectly downregulate many genes involved in DNA repair (e.g., BRCA1, BRCA2, H2AX), apoptosis (neuroepithelial cell transforming 1 (NET1), serine/threonine kinase 17b (STK17B), and in cell cycle regulation (E2F1, CHEK2, and CDK1) [16]. Thus, p21 is a multifaceted protein that is at the center of a complex network of molecular pathways that interacts with several key transcriptional players to regulate the transcription of a broad range of genes. This in turn extends the regulatory function of p21 beyond direct cell cycle control to indirectly regulating cell proliferation, apoptosis, and DNA repair. Importantly, genome-wide expression analysis of normal versus tumor cells would shed light on the multifaceted nature of p21. To explore this nature of p21, and to overcome the limitations of individual studies and their significant variations, bioinformatic tools must be implemented to provide an integrative and more precise and accurate analysis of datasets [138]. Appropriate prediction tools have revolutionized our understanding of the growing complexity of many proteins and may be helpful in unraveling direct and indirect mechanisms mediated by the crosstalk between p21 and various oncogenic and tumorigenic pathways during carcinogenesis.

## 6. Targeting p21 in Cancer Therapeutics

Studies on the role of p21 in cancer therapeutics report contradictory results regarding its role as a mediator of chemosensitivity or chemoresistance. Initial studies have shown that p21 inhibits initiator apoptotic caspase cleavage by TNF-related apoptosis-inducing ligand (DR4/TRAIL) receptor, thus suppressing apoptosis and enhancing survival of human breast and colon cancer cells [139]. It has been suggested that p21 protects human colorectal carcinoma cells from prostaglandin A2-mediated apoptosis by inducing cellular growth arrest [140]. Other studies reported that murine embryonic fibroblasts (MEFs) and HCT-116 cells lacking p21 were sensitive to cisplatin and nitrogen mustard [141,142]. In contrast, augmented p21 expression in lung carcinoma cell line correlated with growth inhibition and enhancement of chemosensitivity to the potent anticancer agent cisplatin [143]. An earlier study has shown that p21 is targeted by miR-33b-3p to promote the survival of A549 human lung cancer cells and their resistance to cisplatin [144]. Furthermore, miR-520g mediated resistance of colorectal cancer cells to 5-fluorouracil (5-FU)- or oxaliplatin-induced apoptosis in vitro and reduced the tumor growth inhibitory effect of 5-FU in vivo partly through downregulating p21 expression [145].

Some anticancer drugs function partly through their ability to induce the expression of p21. For example, histone deacetylase (HDAC) inhibitors induce the expression of p21 through Sp1 binding sites in the p21 promotor, in a p53-independent manner. Trichostatin A promotes p21 expression, and consequently cell cycle arrest and apoptosis, in human gastric carcinoma, oral carcinoma, and multiple myeloma cell lines [146,147]. The HDAC inhibitors, Butyrate and SAHA, induce p21 and apoptosis in human colon cancer and myelomonocytic leukemia cells, respectively [146]. Moreover, fluvastatin and lovastatin act as general HDAC inhibitors and have been shown to induce p21 expression in human cervical HeLa cells [148]. A study has shown that triptolide, a diterpenoid epoxide extracted from the Chinese plant *Tripterygium wilfordii*, upregulated p21 expression and caused G1 phase arrest in colon cancer cells [149]. Similarly, ascochlorin, an isoprenoid antibiotic, induced the expression of p53 and p21 and inhibited colon cancer cell growth by downregulating c-Myc expression [150]. Senescence induced by the DNA-methylating drug temozolomide is dependent on the upregulation of p21 in glioblastoma cells [151]. In addition, small molecule inhibitors of p21 have been devised and include butyrolactone I (BL), sorafenib and UC2288, which is structurally related to sorafenib [72]. BL is a potent inhibitor of p21 that functions by inducing p21 proteasomal degradation in a p53-independent manner. Sorafenib, a tyrosine kinase inhibitor, was effective in reducing p21 levels in renal and hepatocellular carcinoma cells, and its combination with other cytotoxic drugs shows additive effects, suggesting that sorafenib may act as a sensitizing agent for chemotherapeutics used to treat renal cell carcinoma (RCC) by modulating p21 levels. UC2288 significantly reduces cytosolic but not nuclear p21 levels, which is associated with reduced RCC growth, independent of p53 [72]. UC2288 is particularly a promising small inhibitor of p21 which targets oncogenic p21 without affecting global p21 expression levels and without affecting p53 [72]. The use of these drugs may depend on the type of cancer and mode of action of the drug itself. While p21 inducers may serve as promising therapy to treat cancers in which p21 loss correlates with poor prognosis, p21 inhibitors may slow the progression of tumors that upregulate p21.

## 7. Conclusions and Future Directions

The universal CDK inhibitor p21, which was previously thought to play a tumor suppressive role is now well recognized as an important two-faced molecule with remarkable oncogenic activities. This duplicity seems to be based on its role in induction/suppression of apoptosis, its subcellular localization, and is cell type dependent. In addition to controlling cell cycle and promoting cellular senescence, p21 functions as a positive and negative regulator of transcription in response to DNA damage. Given that p21 promotes its effects through the complex interaction with multiple players involved in tumorigenesis, the challenge lies in devising specific therapeutic agents that could inhibit the oncogenic activities of p21 without interfering with its tumor suppressor functions. Therapeutic strategies targeting cytoplasmic p21 may provide a new approach for the treatment of cancer and thus alleviate the resistance to chemotherapy.

## References

[1] Bartek J., Lukas J. (2007). DNA damage checkpoints: From initiation to recovery or adaptation. Curr. Opin. Cell Biol..

[2] Nakanishi M., Shimada M., Niida H. (2006). Genetic instability in cancer cells by impaired cell cycle checkpoints. Cancer Sci..

[3] Eastman A. (2004). Cell cycle checkpoints and their impact on anticancer therapeutic strategies. J. Cell Biochem..

[4] Karimian A., Ahmadi Y., Yousefi B. (2016). Multiple functions of p21 in cell cycle, apoptosis and transcriptional regulation after DNA damage. DNA Repair (Amst.).

[5] Malumbres M., Barbacid M. (2001). To cycle or not to cycle: A critical decision in cancer. Nat. Rev. Cancer.

[6] Ren S., Rollins B.J. (2004). Cyclin C/cdk3 promotes Rb-dependent G0 exit. Cell.

[7] Malumbres M., Barbacid M. (2005). Mammalian cyclin-dependent kinases. Trends Biochem. Sci..

[8] Lim S., Kaldis P. (2013). Cdks, cyclins and CKIs: Roles beyond cell cycle regulation. Development.

[9] Gartel A.L. (2006). Is p21 an oncogene?. Mol. Cancer Ther..

[10] Bertoli C., Skotheim J.M., de Bruin R.A. (2013). Control of cell cycle transcription during G1 and S phases. Nat. Rev. Mol. Cell Biol..

[11] Georgakilas A.G., Martin O.A., Bonner W.M. (2017). p21: A Two-Faced Genome Guardian. Trends Mol. Med..

[12] Niculescu A.B., Chen X., Smeets M., Hengst L., Prives C., Reed S.I. (1998). Effects of p21(Cip1/Waf1) at both the G1/S and the G2/M cell cycle transitions: pRb is a critical determinant in blocking DNA replication and in preventing endoreduplication. Mol. Cell Biol..

[13] Bunz F., Dutriaux A., Lengauer C., Waldman T., Zhou S., Brown J.P., Sedivy J.M., Kinzler K.W., Vogelstein B. (1998). Requirement for p53 and p21 to sustain G2 arrest after DNA damage. Science.

[14] LaBaer J., Garrett M.D., Stevenson L.F., Slingerland J.M., Sandhu C., Chou H.S., Fattaey A., Harlow E. (1997). New functional activities for the p21 family of CDK inhibitors. Genes Dev..

[15] Toufektchan E., Toledo F. (2018). The Guardian of the Genome Revisited: p53 Downregulates Genes Required for Telomere Maintenance, DNA Repair, and Centromere Structure. Cancers.

[16] Engeland K. (2018). Cell cycle arrest through indirect transcriptional repression by p53: I have a DREAM. Cell Death Differ..

[17] Hafner A., Bulyk M.L., Jambhekar A., Lahav G. (2019). The multiple mechanisms that regulate p53 activity and cell fate. Nat. Rev. Mol. Cell Biol..

[18] Chang B.D., Watanabe K., Broude E.V., Fang J., Poole J.C., Kalinichenko T.V., Roninson I.B. (2000). Effects of p21Waf1/Cip1/Sdi1 on cellular gene expression: Implications for carcinogenesis, senescence, and age-related diseases. Proc. Natl. Acad. Sci. USA.

[19] Parveen A., Akash M.S., Rehman K., Kyunn W.W. (2016). Dual Role of p21 in the Progression of Cancer and Its Treatment. Crit. Rev. Eukaryot. Gene Expr..

[20] Jung Y.S., Qian Y., Chen X. (2010). Examination of the expanding pathways for the regulation of p21 expression and activity. Cell Signal..

[21] Harms K., Nozell S., Chen X. (2004). The common and distinct target genes of the p53 family transcription factors. Cell Mol. Life Sci..

[22] Nozell S., Chen X. (2002). p21B, a variant of p21(Waf1/Cip1), is induced by the p53 family. Oncogene.

[23] Gartel A.L., Tyner A.L. (1999). Transcriptional regulation of the p21((WAF1/CIP1)) gene. Exp. Cell Res..

[24] Brooks C.L., Gu W. (2011). The impact of acetylation and deacetylation on the p53 pathway. Protein Cell.

[25] Kastenhuber E.R., Lowe S.W. (2017). Putting p53 in Context. Cell.

[26] Li A.G., Piluso L.G., Cai X., Gadd B.J., Ladurner A.G., Liu X. (2007). An acetylation switch in p53 mediates holo-TFIID recruitment. Mol. Cell.

[27] Beckerman R., Prives C. (2010). Transcriptional regulation by p53. Cold Spring Harb. Perspect. Biol..

[28] Gevry N., Chan H.M., Laflamme L., Livingston D.M., Gaudreau L. (2007). p21 transcription is regulated by differential localization of histone H2A.Z. Genes Dev..

[29] Pauklin S., Kristjuhan A., Maimets T., Jaks V. (2005). ARF and ATM/ATR cooperate in p53-mediated apoptosis upon oncogenic stress. Biochem. Biophys. Res. Commun..

[30] Chai Y.L., Cui J., Shao N., Shyam E., Reddy P., Rao V.N. (1999). The second BRCT domain of BRCA1 proteins interacts with p53 and stimulates transcription from the p21WAF1/CIP1 promoter. Oncogene.

[31] Wulf G.M., Liou Y.C., Ryo A., Lee S.W., Lu K.P. (2002). Role of Pin1 in the regulation of p53 stability and p21 transactivation, and cell cycle checkpoints in response to DNA damage. J. Biol. Chem..

[32] Zacchi P., Gostissa M., Uchida T., Salvagno C., Avolio F., Volinia S., Ronai Z., Blandino G., Schneider C., Del Sal G. (2002). The prolyl isomerase Pin1 reveals a mechanism to control p53 functions after genotoxic insults. Nature.

[33] Tu Y., Wu W., Wu T., Cao Z., Wilkins R., Toh B.H., Cooper M.E., Chai Z. (2007). Antiproliferative autoantigen CDA1 transcriptionally up-regulates p21(Waf1/Cip1) by activating p53 and MEK/ERK1/2 MAPK pathways. J. Biol. Chem..

[34] Rokudai S., Aikawa Y., Tagata Y., Tsuchida N., Taya Y., Kitabayashi I. (2009). Monocytic leukemia zinc finger (MOZ) interacts with p53 to induce p21 expression and cell-cycle arrest. J. Biol. Chem..

[35] Calvisi D.F., Donninger H., Vos M.D., Birrer M.J., Gordon L., Leaner V., Clark G.J. (2009). NORE1A tumor suppressor candidate modulates p21CIP1 via p53. Cancer Res..

[36] Chen X., Johns D.C., Geiman D.E., Marban E., Dang D.T., Hamlin G., Sun R., Yang V.W. (2001). Kruppel-like factor 4 (gut-enriched Kruppel-like factor) inhibits cell proliferation by blocking G1/S progression of the cell cycle. J. Biol. Chem..

[37] Decesse J.T., Medjkane S., Datto M.B., Cremisi C.E. (2001). RB regulates transcription of the p21/WAF1/CIP1 gene. Oncogene.

[38] Abbas T., Dutta A. (2009). p21 in cancer: Intricate networks and multiple activities. Nat. Rev. Cancer.

[39] Xu H., Wang Z., Jin S., Hao H., Zheng L., Zhou B., Zhang W., Lv H., Yuan Y. (2014). Dux4 induces cell cycle arrest at G1 phase through upregulation of p21 expression. Biochem. Biophys. Res. Commun..

[40] Bai Y.Q., Miyake S., Iwai T., Yuasa Y. (2003). CDX2, a homeobox transcription factor, upregulates transcription of the p21/WAF1/CIP1 gene. Oncogene.

[41] Fang Z., Fu Y., Liang Y., Li Z., Zhang W., Jin J., Yang Y., Zha X. (2007). Increased expression of integrin beta1 subunit enhances p21WAF1/Cip1 transcription through the Sp1 sites and p300-mediated histone acetylation in human hepatocellular carcinoma cells. J. Cell Biochem..

[42] Li D., Yea S., Dolios G., Martignetti J.A., Narla G., Wang R., Walsh M.J., Friedman S.L. (2005). Regulation of Kruppel-like factor 6 tumor suppressor activity by acetylation. Cancer Res..

[43] Elston R., Inman G.J. (2012). Crosstalk between p53 and TGF-beta Signalling. J. Signal Transduct..

[44] Oh Y.T., Chun K.H., Park B.D., Choi J.S., Lee S.K. (2007). Regulation of cyclin-dependent kinase inhibitor p21WAF1/CIP1 by protein kinase Cdelta-mediated phosphorylation. Apoptosis.

[45] Scott M.T., Ingram A., Ball K.L. (2002). PDK1-dependent activation of atypical PKC leads to degradation of the p21 tumour modifier protein. EMBO J..

[46] Bornstein G., Bloom J., Sitry-Shevah D., Nakayama K., Pagano M., Hershko A. (2003). Role of the SCFSkp2 ubiquitin ligase in the degradation of p21Cip1 in S phase. J. Biol. Chem..

[47] Amador V., Ge S., Santamaria P.G., Guardavaccaro D., Pagano M. (2007). APC/C(Cdc20) controls the ubiquitin-mediated degradation of p21 in prometaphase. Mol. Cell.

[48] Kim Y., Starostina N.G., Kipreos E.T. (2008). The CRL4Cdt2 ubiquitin ligase targets the degradation of p21Cip1 to control replication licensing. Genes Dev..

[49] Chen X., Chi Y., Bloecher A., Aebersold R., Clurman B.E., Roberts J.M. (2004). N-acetylation and ubiquitin-independent proteasomal degradation of p21(Cip1). Mol. Cell.

[50] Touitou R., Richardson J., Bose S., Nakanishi M., Rivett J., Allday M.J. (2001). A degradation signal located in the C-terminus of p21WAF1/CIP1 is a binding site for the C8 alpha-subunit of the 20S proteasome. EMBO J..

[51] Jin Y., Lee H., Zeng S.X., Dai M.S., Lu H. (2003). MDM2 promotes p21waf1/cip1 proteasomal turnover independently of ubiquitylation. EMBO J..

[52] Deng C., Zhang P., Harper J.W., Elledge S.J., Leder P. (1995). Mice lacking p21CIP1/WAF1 undergo normal development, but are defective in G1 checkpoint control. Cell.

[53] Bhatia K., Fan S., Spangler G., Weintraub M., O’Connor P.M., Judde J.G., Magrath I. (1995). A mutant p21 cyclin-dependent kinase inhibitor isolated from a Burkitt’s lymphoma. Cancer Res..

[54] Vidal M.J., Loganzo F., de Oliveira A.R., Hayward N.K., Albino A.P. (1995). Mutations and defective expression of the WAF1 p21 tumour-suppressor gene in malignant melanomas. Melanoma Res..

[55] Weiss R.H. (2003). p21Waf1/Cip1 as a therapeutic target in breast and other cancers. Cancer Cell.

[56] Akhter N., Akhtar M.S., Ahmad M.M., Haque S., Siddiqui S., Hasan S.I., Shukla N.K., Husain S.A. (2014). Association of mutation and hypermethylation of p21 gene with susceptibility to breast cancer: A study from north India. Mol. Biol. Rep..

[57] Gartel A.L., Shchors K. (2003). Mechanisms of c-myc-mediated transcriptional repression of growth arrest genes. Exp. Cell Res..

[58] Mukherjee S., Conrad S.E. (2005). c-Myc suppresses p21WAF1/CIP1 expression during estrogen signaling and antiestrogen resistance in human breast cancer cells. J. Biol. Chem..

[59] Jung P., Hermeking H. (2009). The c-MYC-AP4-p21 cascade. Cell Cycle.

[60] Van de Wetering M., Sancho E., Verweij C., de Lau W., Oving I., Hurlstone A., van der Horn K., Batlle E., Coudreuse D., Haramis A.P. (2002). The beta-catenin/TCF-4 complex imposes a crypt progenitor phenotype on colorectal cancer cells. Cell.

[61] Jung P., Menssen A., Mayr D., Hermeking H. (2008). AP4 encodes a c-MYC-inducible repressor of p21. Proc. Natl. Acad. Sci. USA.

[62] Prince S., Carreira S., Vance K.W., Abrahams A., Goding C.R. (2004). Tbx2 directly represses the expression of the p21(WAF1) cyclin-dependent kinase inhibitor. Cancer Res..

[63] Kang Z.H., Wang C.Y., Zhang W.L., Zhang J.T., Yuan C.H., Zhao P.W., Lin Y.Y., Hong S., Li C.Y., Wang L. (2014). Histone deacetylase HDAC4 promotes gastric cancer SGC-7901 cells progression via p21 repression. PLoS ONE.

[64] Zhang Q., Song Y., Chen W., Wang X., Miao Z., Cao L., Li F., Wang G. (2015). By recruiting HDAC1, MORC2 suppresses p21 Waf1/Cip1 in gastric cancer. Oncotarget.

[65] Qiu L., Wu J., Pan C., Tan X., Lin J., Liu R., Chen S., Geng R., Huang W. (2016). Downregulation of CDC27 inhibits the proliferation of colorectal cancer cells via the accumulation of p21Cip1/Waf1. Cell Death Dis.

[66] Abbas T., Sivaprasad U., Terai K., Amador V., Pagano M., Dutta A. (2008). PCNA-dependent regulation of p21 ubiquitylation and degradation via the CRL4Cdt2 ubiquitin ligase complex. Genes Dev..

[67] Stuart S.A., Wang J.Y. (2009). Ionizing radiation induces ATM-independent degradation of p21Cip1 in transformed cells. J. Biol. Chem..

[68] Blandino G., Di Agostino S. (2018). New therapeutic strategies to treat human cancers expressing mutant p53 proteins. J. Exp. Clin. Cancer Res..

[69] Thakur V.S., Ruhul Amin A.R., Paul R.K., Gupta K., Hastak K., Agarwal M.K., Jackson M.W., Wald D.N., Mukhtar H., Agarwal M.L. (2010). p53-Dependent p21-mediated growth arrest pre-empts and protects HCT116 cells from PUMA-mediated apoptosis induced by EGCG. Cancer Lett..

[70] Larrieu D., Ythier D., Brambilla C., Pedeux R. (2010). ING2 controls the G1 to S-phase transition by regulating p21 expression. Cell Cycle.

[71] Gartel A.L., Najmabadi F., Goufman E., Tyner A.L. (2000). A role for E2F1 in Ras activation of p21(WAF1/CIP1) transcription. Oncogene.

[72] Liu R., Wettersten H.I., Park S.H., Weiss R.H. (2013). Small-molecule inhibitors of p21 as novel therapeutics for chemotherapy-resistant kidney cancer. Future Med. Chem..

[73] Romanov V.S., Rudolph K.L. (2016). p21 shapes cancer evolution. Nat. Cell Biol..

[74] Gawriluk T.R., Simkin J., Thompson K.L., Biswas S.K., Clare-Salzler Z., Kimani J.M., Kiama S.G., Smith J.J., Ezenwa V.O., Seifert A.W. (2016). Comparative analysis of ear-hole closure identifies epimorphic regeneration as a discrete trait in mammals. Nat. Commun..

[75] Winters Z.E., Hunt N.C., Bradburn M.J., Royds J.A., Turley H., Harris A.L., Norbury C.J. (2001). Subcellular localisation of cyclin B, Cdc2 and p21(WAF1/CIP1) in breast cancer. association with prognosis. Eur. J. Cancer.

[76] Ohata M., Nakamura S., Fujita H., Isemura M. (2005). Prognostic implications of p21 (Waf1/Cip1) immunolocalization in multiple myeloma. Biomed. Res..

[77] Adams P.D., Li X., Sellers W.R., Baker K.B., Leng X., Harper J.W., Taya Y., Kaelin W.G. (1999). Retinoblastoma protein contains a C-terminal motif that targets it for phosphorylation by cyclin-cdk complexes. Mol. Cell Biol..

[78] Satyanarayana A., Hilton M.B., Kaldis P. (2008). p21 Inhibits Cdk1 in the absence of Cdk2 to maintain the G1/S phase DNA damage checkpoint. Mol. Biol. Cell.

[79] Gulbis J.M., Kelman Z., Hurwitz J., O’Donnell M., Kuriyan J. (1996). Structure of the C-terminal region of p21(WAF1/CIP1) complexed with human PCNA. Cell.

[80] Gottifredi V., McKinney K., Poyurovsky M.V., Prives C. (2004). Decreased p21 levels are required for efficient restart of DNA synthesis after S phase block. J. Biol. Chem..

[81] Cazzalini O., Scovassi A.I., Savio M., Stivala L.A., Prosperi E. (2010). Multiple roles of the cell cycle inhibitor p21(CDKN1A) in the DNA damage response. Mutat. Res..

[82] Smits V.A., Klompmaker R., Vallenius T., Rijksen G., Makela T.P., Medema R.H. (2000). p21 inhibits Thr161 phosphorylation of Cdc2 to enforce the G2 DNA damage checkpoint. J. Biol. Chem..

[83] Kreis N.N., Sanhaji M., Rieger M.A., Louwen F., Yuan J. (2014). p21Waf1/Cip1 deficiency causes multiple mitotic defects in tumor cells. Oncogene.

[84] Welcker M., Lukas J., Strauss M., Bartek J. (1998). p21WAF1/CIP1 mutants deficient in inhibiting cyclin-dependent kinases (CDKs) can promote assembly of active cyclin D/CDK4(6) complexes in human tumor cells. Cancer Res..

[85] Cheng M., Olivier P., Diehl J.A., Fero M., Roussel M.F., Roberts J.M., Sherr C.J. (1999). The p21(Cip1) and p27(Kip1) CDK ’inhibitors’ are essential activators of cyclin D-dependent kinases in murine fibroblasts. EMBO J..

[86] Yue F., Cheng Y., Breschi A., Vierstra J., Wu W., Ryba T., Sandstrom R., Ma Z., Davis C., Pope B.D. (2014). A comparative encyclopedia of DNA elements in the mouse genome. Nature.

[87] Deng T., Yan G., Song X., Xie L., Zhou Y., Li J., Hu X., Li Z., Hu J., Zhang Y. (2018). Deubiquitylation and stabilization of p21 by USP11 is critical for cell-cycle progression and DNA damage responses. Proc. Natl. Acad. Sci. USA.

[88] Gartel A.L., Tyner A.L. (2002). The role of the cyclin-dependent kinase inhibitor p21 in apoptosis. Mol. Cancer Ther..

[89] Suzuki A., Tsutomi Y., Miura M., Akahane K. (1999). Caspase 3 inactivation to suppress Fas-mediated apoptosis: Identification of binding domain with p21 and ILP and inactivation machinery by p21. Oncogene.

[90] Gervais J.L., Seth P., Zhang H. (1998). Cleavage of CDK inhibitor p21(Cip1/Waf1) by caspases is an early event during DNA damage-induced apoptosis. J. Biol. Chem..

[91] Asada M., Yamada T., Ichijo H., Delia D., Miyazono K., Fukumuro K., Mizutani S. (1999). Apoptosis inhibitory activity of cytoplasmic p21(Cip1/WAF1) in monocytic differentiation. EMBO J..

[92] Baptiste-Okoh N., Barsotti A.M., Prives C. (2008). Caspase 2 is both required for p53-mediated apoptosis and downregulated by p53 in a p21-dependent manner. Cell Cycle.

[93] Zhang Y., Fujita N., Tsuruo T. (1999). Caspase-mediated cleavage of p21Waf1/Cip1 converts cancer cells from growth arrest to undergoing apoptosis. Oncogene.

[94] Kaneuchi M., Yamashita T., Shindoh M., Segawa K., Takahashi S., Furuta I., Fujimoto S., Fujinaga K. (1999). Induction of apoptosis by the p53-273L (Arg --> Leu) mutant in HSC3 cells without transactivation of p21Waf1/Cip1/Sdi1 and bax. Mol. Carcinog..

[95] Shaulian E., Schreiber M., Piu F., Beeche M., Wagner E.F., Karin M. (2000). The mammalian UV response: C-Jun induction is required for exit from p53-imposed growth arrest. Cell.

[96] Canman C.E., Gilmer T.M., Coutts S.B., Kastan M.B. (1995). Growth factor modulation of p53-mediated growth arrest versus apoptosis. Genes Dev..

[97] Helt C.E., Rancourt R.C., Staversky R.J., O’Reilly M.A. (2001). p53-dependent induction of p21(Cip1/WAF1/Sdi1) protects against oxygen-induced toxicity. Toxicol. Sci..

[98] Li C.Y., Suardet L., Little J.B. (1995). Potential role of WAF1/Cip1/p21 as a mediator of TGF-beta cytoinhibitory effect. J. Biol. Chem..

[99] Gartel A.L. (2005). The conflicting roles of the cdk inhibitor p21(CIP1/WAF1) in apoptosis. Leuk. Res..

[100] Qiao L., McKinstry R., Gupta S., Gilfor D., Windle J.J., Hylemon P.B., Grant S., Fisher P.B., Dent P. (2002). Cyclin kinase inhibitor p21 potentiates bile acid-induced apoptosis in hepatocytes that is dependent on p53. Hepatology.

[101] Kang K.H., Kim W.H., Choi K.H. (1999). p21 promotes ceramide-induced apoptosis and antagonizes the antideath effect of Bcl-2 in human hepatocarcinoma cells. Exp. Cell Res..

[102] Hingorani R., Bi B., Dao T., Bae Y., Matsuzawa A., Crispe I.N. (2000). CD95/Fas signaling in T lymphocytes induces the cell cycle control protein p21cip-1/WAF-1, which promotes apoptosis. J. Immunol..

[103] Ghanem L., Steinman R. (2005). A proapoptotic function of p21 in differentiating granulocytes. Leuk. Res..

[104] Soria G., Speroni J., Podhajcer O.L., Prives C., Gottifredi V. (2008). p21 differentially regulates DNA replication and DNA-repair-associated processes after UV irradiation. J. Cell Sci..

[105] Soria G., Podhajcer O., Prives C., Gottifredi V. (2006). P21Cip1/WAF1 downregulation is required for efficient PCNA ubiquitination after UV irradiation. Oncogene.

[106] Cazzalini O., Perucca P., Savio M., Necchi D., Bianchi L., Stivala L.A., Ducommun B., Scovassi A.I., Prosperi E. (2008). Interaction of p21(CDKN1A) with PCNA regulates the histone acetyltransferase activity of p300 in nucleotide excision repair. Nucleic Acids Res..

[107] Moldovan G.L., Pfander B., Jentsch S. (2007). PCNA, the maestro of the replication fork. Cell.

[108] Fotedar R., Bendjennat M., Fotedar A. (2004). Role of p21WAF1 in the cellular response to UV. Cell Cycle.

[109] Gratchev A. (2008). The nucleotide excision repair of DNA in human cells and its association with xeroderma pigmentosum. Adv. Exp. Med. Biol..

[110] Stoyanova T., Yoon T., Kopanja D., Mokyr M.B., Raychaudhuri P. (2008). The xeroderma pigmentosum group E gene product DDB2 activates nucleotide excision repair by regulating the level of p21Waf1/Cip1. Mol. Cell Biol..

[111] Stivala L.A., Riva F., Cazzalini O., Savio M., Prosperi E. (2001). p21(waf1/cip1)-null human fibroblasts are deficient in nucleotide excision repair downstream the recruitment of PCNA to DNA repair sites. Oncogene.

[112] Tillhon M., Cazzalini O., Nardo T., Necchi D., Sommatis S., Stivala L.A., Scovassi A.I., Prosperi E. (2012). p300/CBP acetyl transferases interact with and acetylate the nucleotide excision repair factor XPG. DNA Repair (Amst.).

[113] Jakob B., Scholz M., Taucher-Scholz G. (2002). Characterization of CDKN1A (p21) binding to sites of heavy-ion-induced damage: Colocalization with proteins involved in DNA repair. Int. J. Radiat. Biol..

[114] Mauro M., Rego M.A., Boisvert R.A., Esashi F., Cavallo F., Jasin M., Howlett N.G. (2012). p21 promotes error-free replication-coupled DNA double-strand break repair. Nucleic Acids Res..

[115] Koike M., Yutoku Y., Koike A. (2011). Accumulation of p21 proteins at DNA damage sites independent of p53 and core NHEJ factors following irradiation. Biochem. Biophys. Res. Commun..

[116] Yaglom J.A., McFarland C., Mirny L., Sherman M.Y. (2014). Oncogene-triggered suppression of DNA repair leads to DNA instability in cancer. Oncotarget.

[117] Mei S., Flemington E.K., Zhang K. (2017). A computational framework for distinguishing direct versus indirect interactions in human functional protein-protein interaction networks. Integr. Biol. (Camb.).

[118] Perkins N.D. (2002). Not just a CDK inhibitor: Regulation of transcription by p21(WAF1/CIP1/SDI1). Cell Cycle.

[119] Perkins N.D., Felzien L.K., Betts J.C., Leung K., Beach D.H., Nabel G.J. (1997). Regulation of NF-kappaB by cyclin-dependent kinases associated with the p300 coactivator. Science.

[120] Redeuilh G., Attia A., Mester J., Sabbah M. (2002). Transcriptional activation by the oestrogen receptor alpha is modulated through inhibition of cyclin-dependent kinases. Oncogene.

[121] Coqueret O. (2003). New roles for p21 and p27 cell-cycle inhibitors: A function for each cell compartment?. Trends Cell Biol..

[122] Fritah A., Saucier C., Mester J., Redeuilh G., Sabbah M. (2005). p21WAF1/CIP1 selectively controls the transcriptional activity of estrogen receptor alpha. Mol. Cell Biol..

[123] Broude E.V., Demidenko Z.N., Vivo C., Swift M.E., Davis B.M., Blagosklonny M.V., Roninson I.B. (2007). p21 (CDKN1A) is a negative regulator of p53 stability. Cell Cycle.

[124] Coqueret O., Gascan H. (2000). Functional interaction of STAT3 transcription factor with the cell cycle inhibitor p21WAF1/CIP1/SDI1. J. Biol. Chem..

[125] Delavaine L., La Thangue N.B. (1999). Control of E2F activity by p21Waf1/Cip1. Oncogene.

[126] Kitaura H., Shinshi M., Uchikoshi Y., Ono T., Iguchi-Ariga S.M., Ariga H. (2000). Reciprocal regulation via protein-protein interaction between c-Myc and p21(cip1/waf1/sdi1) in DNA replication and transcription. J. Biol. Chem..

[127] Vigneron A., Cherier J., Barre B., Gamelin E., Coqueret O. (2006). The cell cycle inhibitor p21waf1 binds to the myc and cdc25A promoters upon DNA damage and induces transcriptional repression. J. Biol. Chem..

[128] Dai M., Al-Odaini A.A., Arakelian A., Rabbani S.A., Ali S., Lebrun J.J. (2012). A novel function for p21Cip1 and acetyltransferase p/CAF as critical transcriptional regulators of TGFbeta-mediated breast cancer cell migration and invasion. Breast Cancer Res..

[129] Zhu H., Chang B.D., Uchiumi T., Roninson I.B. (2002). Identification of promoter elements responsible for transcriptional inhibition of polo-like kinase 1 and topoisomerase IIalpha genes by p21(WAF1/CIP1/SDI1). Cell Cycle.

[130] Ferrandiz N., Caraballo J.M., Garcia-Gutierrez L., Devgan V., Rodriguez-Paredes M., Lafita M.C., Bretones G., Quintanilla A., Munoz-Alonso M.J., Blanco R. (2012). p21 as a transcriptional co-repressor of S-phase and mitotic control genes. PLoS ONE.

[131] Wang J., Devgan V., Corrado M., Prabhu N.S., El-Deiry W.S., Riccardi C., Pandolfi P.P., Missero C., Dotto G.P. (2005). Glucocorticoid-induced tumor necrosis factor receptor is a p21Cip1/WAF1 transcriptional target conferring resistance of keratinocytes to UV light-induced apoptosis. J. Biol. Chem..

[132] Devgan V., Mammucari C., Millar S.E., Brisken C., Dotto G.P. (2005). p21WAF1/Cip1 is a negative transcriptional regulator of Wnt4 expression downstream of Notch1 activation. Genes Dev..

[133] Gregory D.J., Garcia-Wilson E., Poole J.C., Snowden A.W., Roninson I.B., Perkins N.D. (2002). Induction of transcription through the p300 CRD1 motif by p21WAF1/CIP1 is core promoter specific and cyclin dependent kinase independent. Cell Cycle.

[134] Tan H.H., Porter A.G. (2009). p21(WAF1) negatively regulates DNMT1 expression in mammalian cells. Biochem. Biophys. Res. Commun..

[135] Kim H.K., Kang M.A., Kim M.S., Shin Y.J., Chi S.G., Jeong J.H. (2018). Transcriptional Repression of High-Mobility Group Box 2 by p21 in Radiation-Induced Senescence. Mol. Cells.

[136] Trakala M., Arias C.F., Garcia M.I., Moreno-Ortiz M.C., Tsilingiri K., Fernandez P.J., Mellado M., Diaz-Meco M.T., Moscat J., Serrano M. (2009). Regulation of macrophage activation and septic shock susceptibility via p21(WAF1/CIP1). Eur. J. Immunol..

[137] Yao H., Yang S.R., Edirisinghe I., Rajendrasozhan S., Caito S., Adenuga D., O’Reilly M.A., Rahman I. (2008). Disruption of p21 attenuates lung inflammation induced by cigarette smoke, LPS, and fMLP in mice. Am. J. Respir. Cell Mol. Biol..

[138] Lapatas V., Stefanidakis M., Jimenez R.C., Via A., Schneider M.V. (2015). Data integration in biological research: An overview. J. Biol. Res. (Thessalon.).

[139] Xu S.Q., El-Deiry W.S. (2000). p21(WAF1/CIP1) inhibits initiator caspase cleavage by TRAIL death receptor DR4. Biochem. Biophys. Res. Commun..

[140] Gorospe M., Wang X., Guyton K.Z., Holbrook N.J. (1996). Protective role of p21(Waf1/Cip1) against prostaglandin A2-mediated apoptosis of human colorectal carcinoma cells. Mol. Cell Biol..

[141] Fan S., Chang J.K., Smith M.L., Duba D., Fornace A.J., O’Connor P.M. (1997). Cells lacking CIP1/WAF1 genes exhibit preferential sensitivity to cisplatin and nitrogen mustard. Oncogene.

[142] McDonald E.R., Wu G.S., Waldman T., El-Deiry W.S. (1996). Repair Defect in p21 WAF1/CIP1 -/- human cancer cells. Cancer Res..

[143] Wei J., Zhao J., Long M., Han Y., Wang X., Lin F., Ren J., He T., Zhang H. (2010). p21WAF1/CIP1 gene transcriptional activation exerts cell growth inhibition and enhances chemosensitivity to cisplatin in lung carcinoma cell. BMC Cancer.

[144] Xu S., Huang H., Chen Y.N., Deng Y.T., Zhang B., Xiong X.D., Yuan Y., Zhu Y., Huang H., Xie L. (2016). DNA damage responsive miR-33b-3p promoted lung cancer cells survival and cisplatin resistance by targeting p21(WAF1/CIP1). Cell Cycle.

[145] Zhang Y., Geng L., Talmon G., Wang J. (2015). MicroRNA-520g confers drug resistance by regulating p21 expression in colorectal cancer. J. Biol. Chem..

[146] Eckschlager T., Plch J., Stiborova M., Hrabeta J. (2017). Histone Deacetylase Inhibitors as Anticancer Drugs. Int. J. Mol. Sci..

[147] Geng Y., Liu J., Xie Y., Jiang H., Zuo K., Li T., Liu Z. (2018). Trichostatin A promotes GLI1 degradation and P21 expression in multiple myeloma cells. Cancer Manag. Res..

[148] Lin C.K., Liu S.T., Chang C.C., Huang S.M. (2019). Regulatory mechanisms of fluvastatin and lovastatin for the p21 induction in human cervical cancer HeLa cells. PLoS ONE.

[149] Liu J., Shen M., Yue Z., Yang Z., Wang M., Li C., Xin C., Wang Y., Mei Q., Wang Z. (2012). Triptolide inhibits colon-rectal cancer cells proliferation by induction of G1 phase arrest through upregulation of p21. Phytomedicine.

[150] Jeong Y.J., Hoe H.S., Cho H.J., Park K.K., Kim D.D., Kim C.H., Magae J., Kang D.W., Lee S.R., Chang Y.C. (2018). Suppression of c-Myc enhances p21(WAF1/CIP1) -mediated G1 cell cycle arrest through the modulation of ERK phosphorylation by ascochlorin. J. Cell Biochem..

[151] Aasland D., Gotzinger L., Hauck L., Berte N., Meyer J., Effenberger M., Schneider S., Reuber E.E., Roos W.P., Tomicic M.T. (2019). Temozolomide Induces Senescence and Repression of DNA Repair Pathways in Glioblastoma Cells via Activation of ATR-CHK1, p21, and NF-kappaB. Cancer Res..

